# Supplementary material for: Dynamic composition of stress granules in Trypanosoma brucei
Source: PLoS Pathog. 2024 Oct 31;20(10):e1012666. doi: 10.1371/journal.ppat.1012666 (PMC11556693; doi:10.1371/journal.ppat.1012666)
Supplement: S1 Table — (PDF) [file ppat.1012666.s001.pdf]

Supplementary Table 1. Composition of medium used in this study. Components that are not included in the respective medium are noted by (X).

	Chemical name	Cunningham	Energy-depleted
Inorganic salts	NaH_2PO_4	0.53	0.53
	$MgSO_4$	1.8	1.8
	KCl	2.98	2.98
	$CaCl_2.2H_2O$	0.15	0.15
Sugars	Glucose	0.7	**X**
	Fructose	0.4	**X**
	Sucrose	0.4	**X**
Organic acids	L-malic acid	0.67	**X**
	alpha-ketoglutarate	0.37	**X**
	Fumaric acid	0.055	**X**
	Succinic acid	0.06	**X**
Amino acids	Beta alanine	0.5	0.5
	DL alanine	1.09	1.09
	L Arginine	0.44	0.44
	L Aspartic acid	0.11	0.11
	L Cysteine HCl	0.08	0.08
	L Cystine	0.03	0.03
	L Glutamic acid	0.25	0.25
	Glycine	0.12	0.12
	L Histidine	0.16	0.16
	DL Isoleucine	0.09	0.09
	L Leucine	0.09	0.09
	L Lysine	0.15	0.15
	DL Methionine	0.2	0.2
	L Phenylalanine	0.2	0.2
	L Proline	1	**X**
	L Serine	0.2	0.2
	Taurine	0.27	0.27
	L Tryptophan	0.1	0.1
	L Tyrosine	0.2	0.2
	DL Valine	0.21	0.21
	L Asparagine	0.24	0.24
	L Threonine	0.1	**X**
Others	HEPES	6	6
	Sodium bicarbonate	2	2
	BME vitamin mixture	0.20%	0.20%
	Phenol red (0.5%)	0.40%	0.40%
	FBS	10%	**X**
