# Supplementary material for: Dynamic composition of stress granules in Trypanosoma brucei
Source: PLoS Pathog. 2024 Oct 31;20(10):e1012666. doi: 10.1371/journal.ppat.1012666 (PMC11556693; doi:10.1371/journal.ppat.1012666)
Supplement: S2 Table — (PDF) [file ppat.1012666.s002.pdf]

Supplementary Table 2. List of plasmids used in this study.

Plasmid name	Backbone	Selection drug	Insert description	Insert Map	GeneID
pMO-PABP2-mScarlet	pMOTag	Puromycin	PABP2 3'CDS (1360-1665) and PABP2 3'UTR (339 bp)	AvrII-3'CDS-TY-mScarlet-PuroR-3'UTR-SpeI	Tb927.9.10770
pMO-PABP2-HA	pMOTag	Neomycin	PABP2 3'CDS (1360-1665) and PABP2 3'UTR (339 bp)	AvrII-3'CDS-HA-NeoR-3'UTR-SpeI	Tb927.9.10770
pMO-PABP2-TY-GFP	pMOTag	Blasticidine	PABP2 3'CDS (1360-1665) and PABP2 3'UTR (339 bp)	AvrII-3'CDS-TY-GFP-BlaR-3'UTR-SpeI	Tb927.9.10770
pMO-mNeonGreen-TY-DHH1	pMOTag	Hygromycin	DHH1 5'UTR (278) and DHH1 5'CDS (1-523)	AvrII-5'UTR-AvrII-HygR-mNeonGreen-TY-XbaI-5'CDS-XbaI	Tb927.10.3990
pMO-SCD6-mScarlet	pMOTag	Puromycin	SCD6 3'CDS (496-840) and SCD6 3'UTR (314 bp)	AvrII-3'CDS-TY-mScarlet-PuroR-3'UTR-SpeI	Tb927.11.550
pMO-ALPH1-mScarlet	pMOTag	Blasticidine	ALPH1 3'CDS (1807-2202) and ALPH1 3'UTR (429 bp)	AvrII-3'CDS-TY-mSCarlet-BlaR-3'UTR-SpeI	Tb927.6.640
pMO-CAF1-mScarlet	pMOTag	Blasticidine	CAF1 3'CDS (664-1053) and CAF1 3'UTR (501 bp)	AvrII-3'CDS-TY-mSCarlet-BlaR-3'UTR-SpeI	Tb927.6.600
pMO-eIF4E1-mScarlet	pMOTag	Blasticidine	eIF4E1 3'CDS (1-699) and eIF4E1 3'UTR (354 bp)	AvrII-3'CDS-TY-mSCarlet-BlaR-3'UTR-SpeI	Tb927.11.2260
pMO-eIF4G1-mScarlet	pMOTag	Blasticidine	eIF4G1 3'CDS (3109-3354) and eIF4G1 3'UTR (246 bp)	AvrII-3'CDS-TY-mSCarlet-BlaR-3'UTR-SpeI	Tb927.5.1490
pMO-mScarlet-RBP6	pMOTag	Blasticidine	RBP6 5'UTR (311 bp) and RBP6 5'CDS (1-720)	AvrII-5'UTR-BlaR-mScarlet-TY-5'CDS-XbaI	Tb927.3.2930
pMO-ALPH1-HA	pMOTag	Neomycin	ALPH1 3'CDS (1807-2202) and ALPH1 3'UTR (429 bp)	AvrII-3'CDS-HA-NeoR-3'UTR-SpeI	Tb927.6.640
