# Supplementary figures and images for: Dynamic composition of stress granules in Trypanosoma brucei
Source: PLoS Pathog. 2024 Oct 31;20(10):e1012666. doi: 10.1371/journal.ppat.1012666 (PMC11556693; doi:10.1371/journal.ppat.1012666)

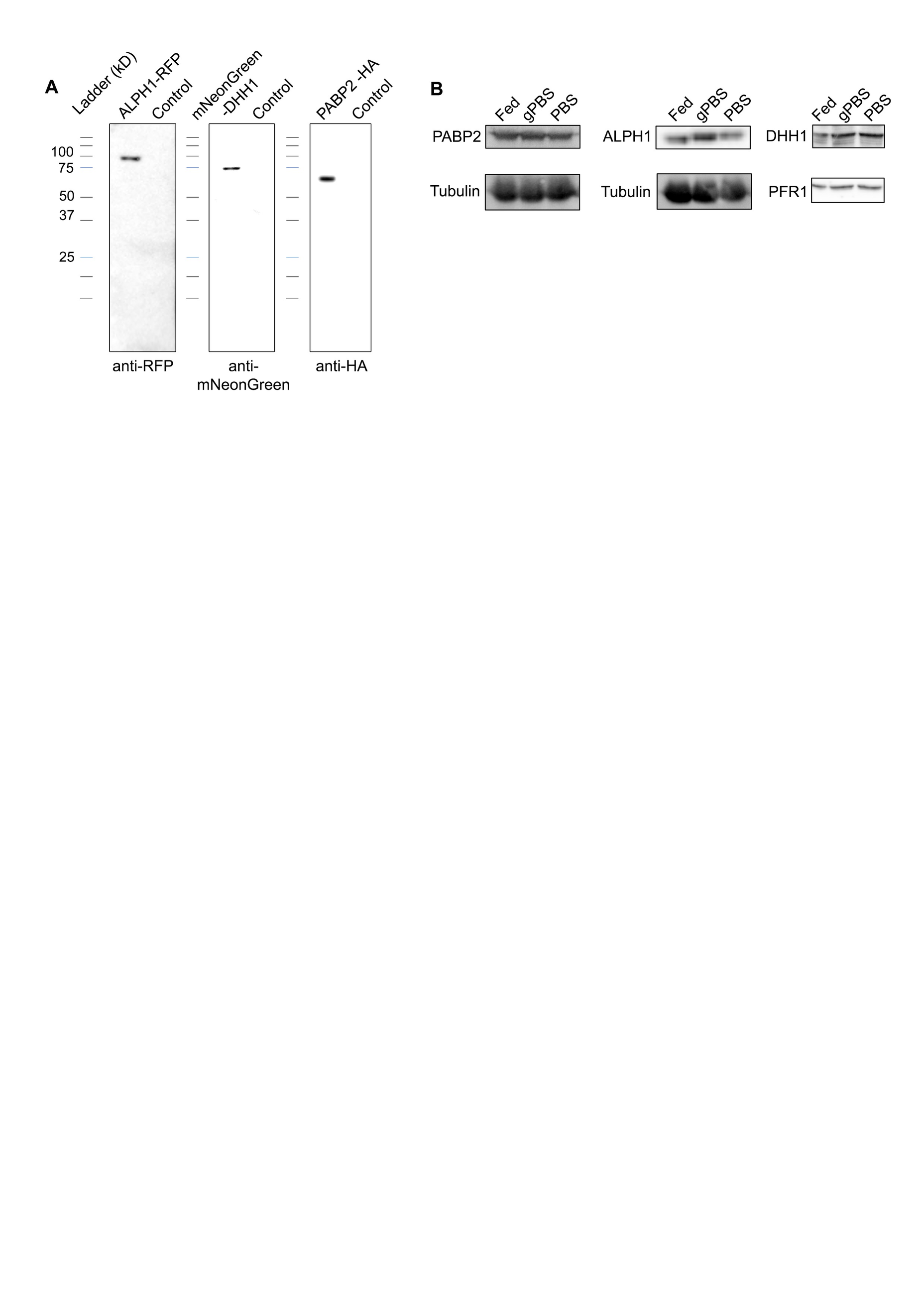

Supplement: S1 Fig — (TIF) [file ppat.1012666.s003.tif]

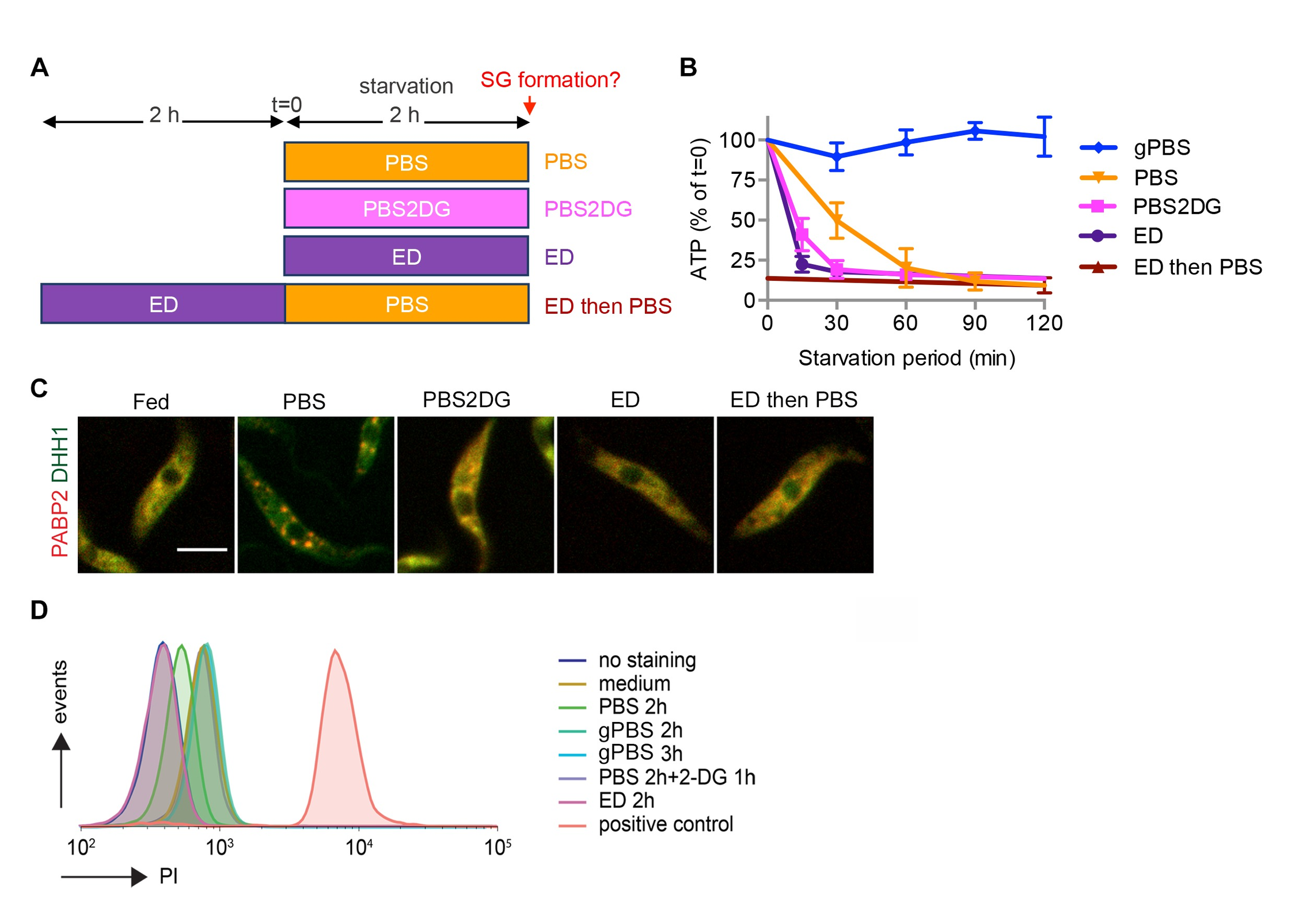

Supplement: S2 Fig — (TIF) [file ppat.1012666.s004.tif]

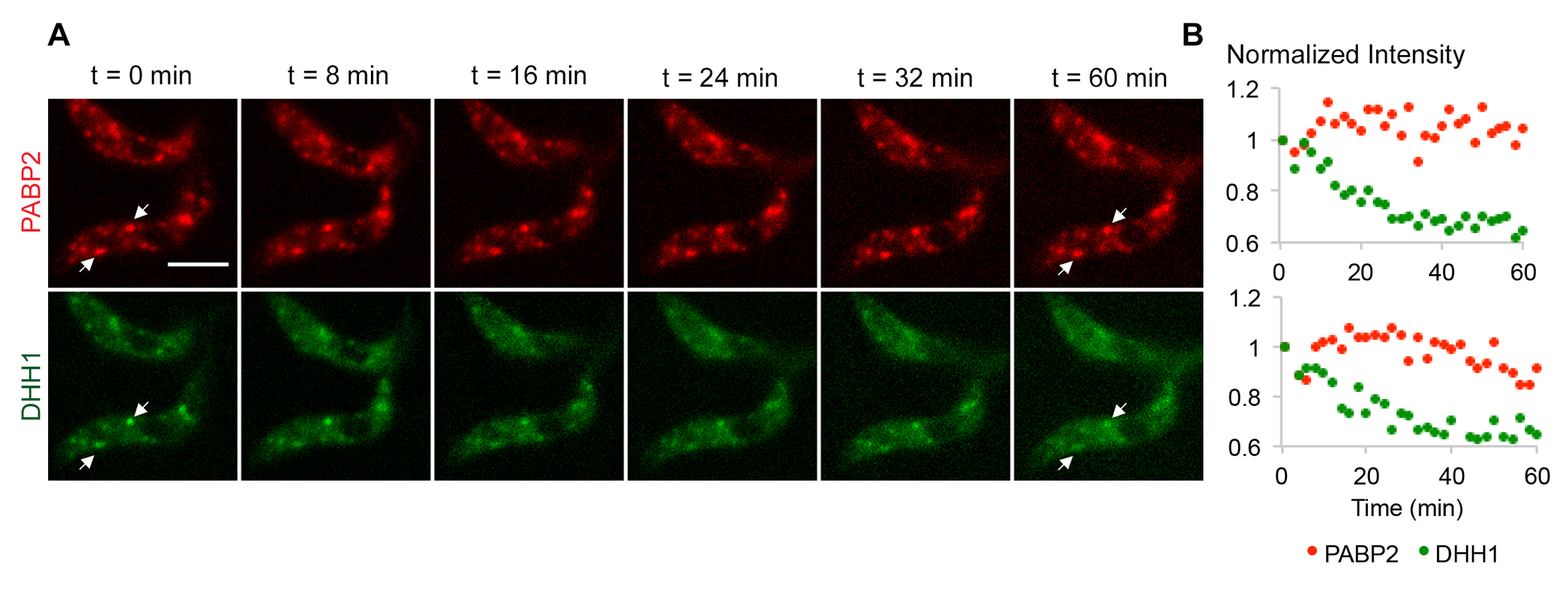

Supplement: S3 Fig — (TIF) [file ppat.1012666.s005.tif]

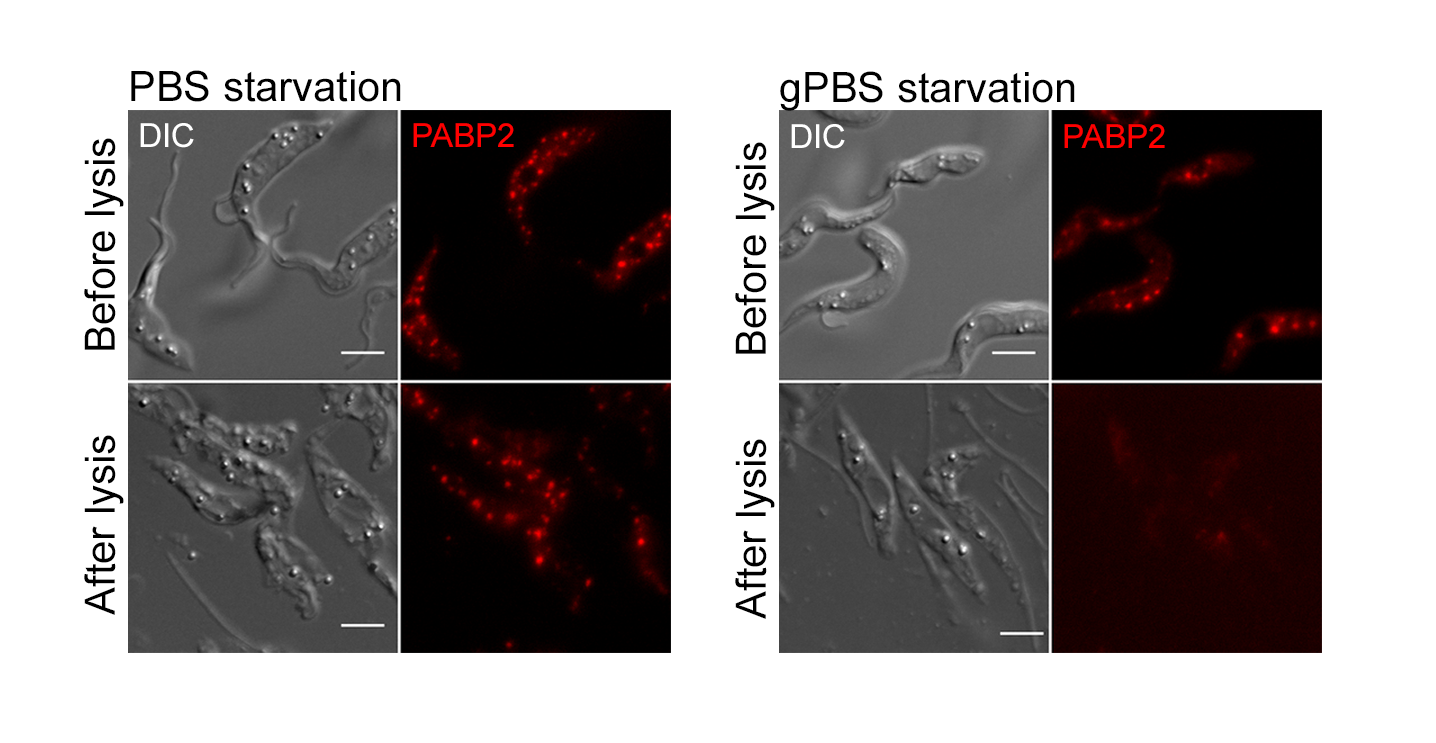

Supplement: S4 Fig — (TIF) [file ppat.1012666.s006.tif]

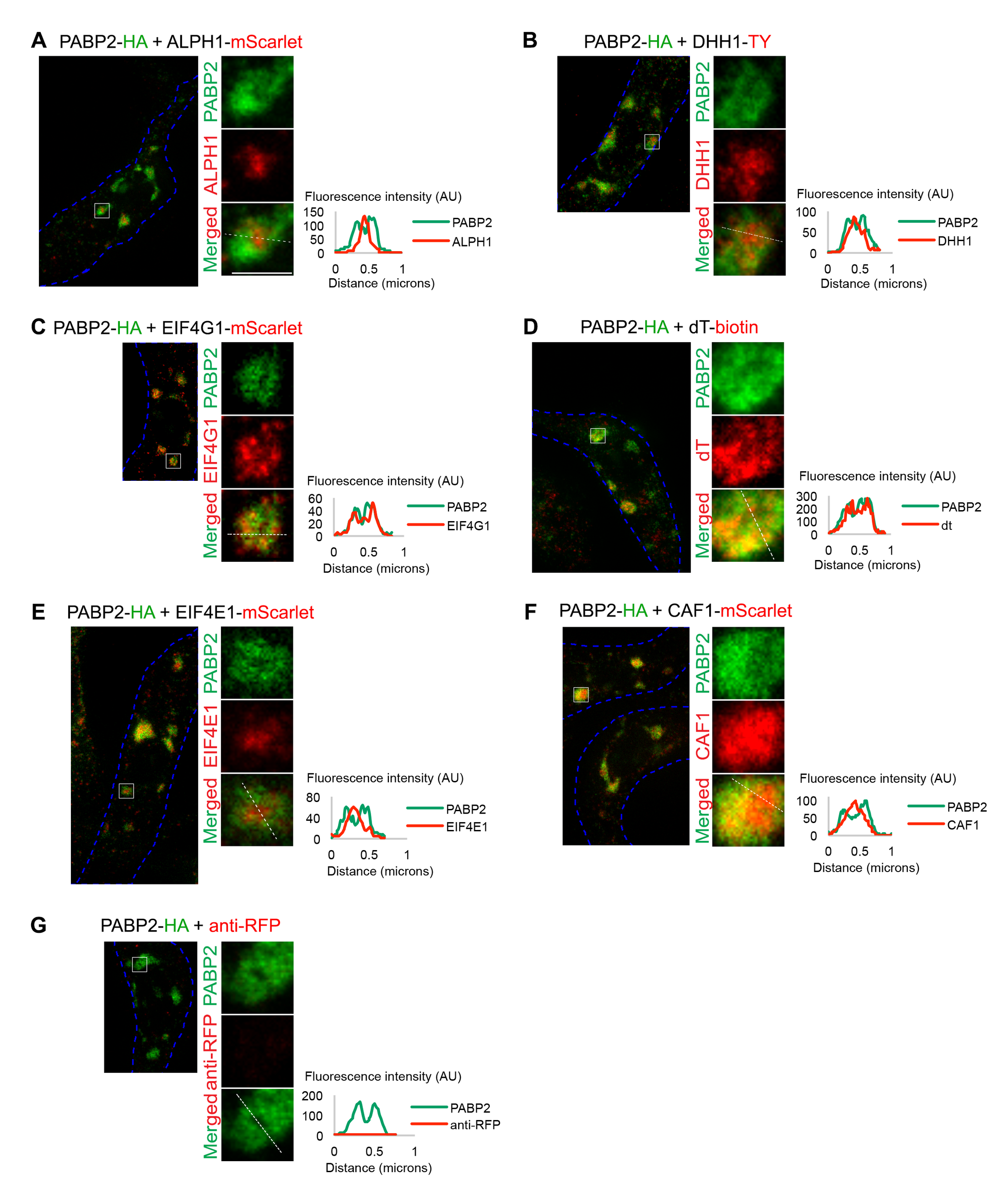

Supplement: S5 Fig — (TIF) [file ppat.1012666.s007.tif]

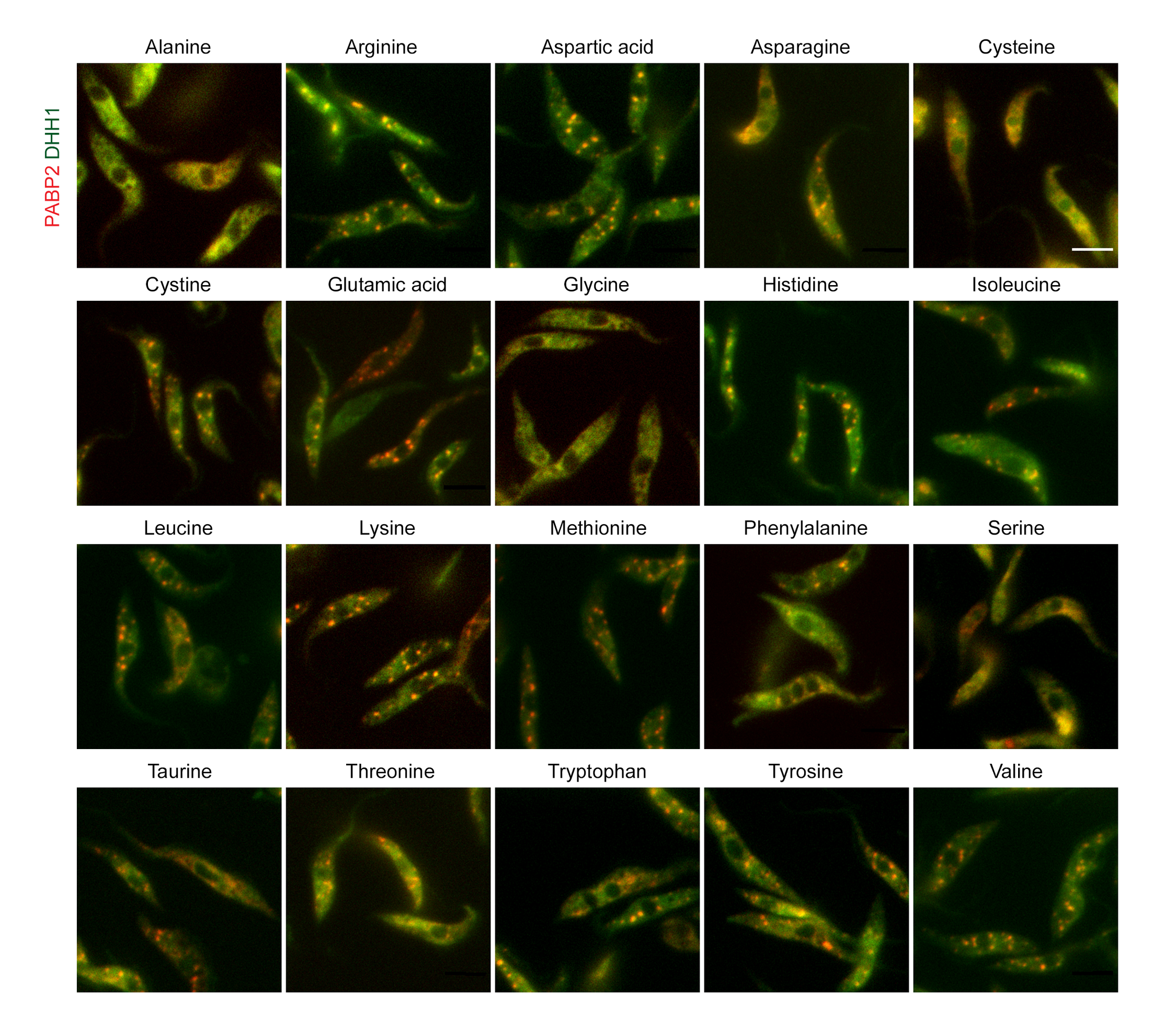

Supplement: S6 Fig — (TIF) [file ppat.1012666.s008.tif]

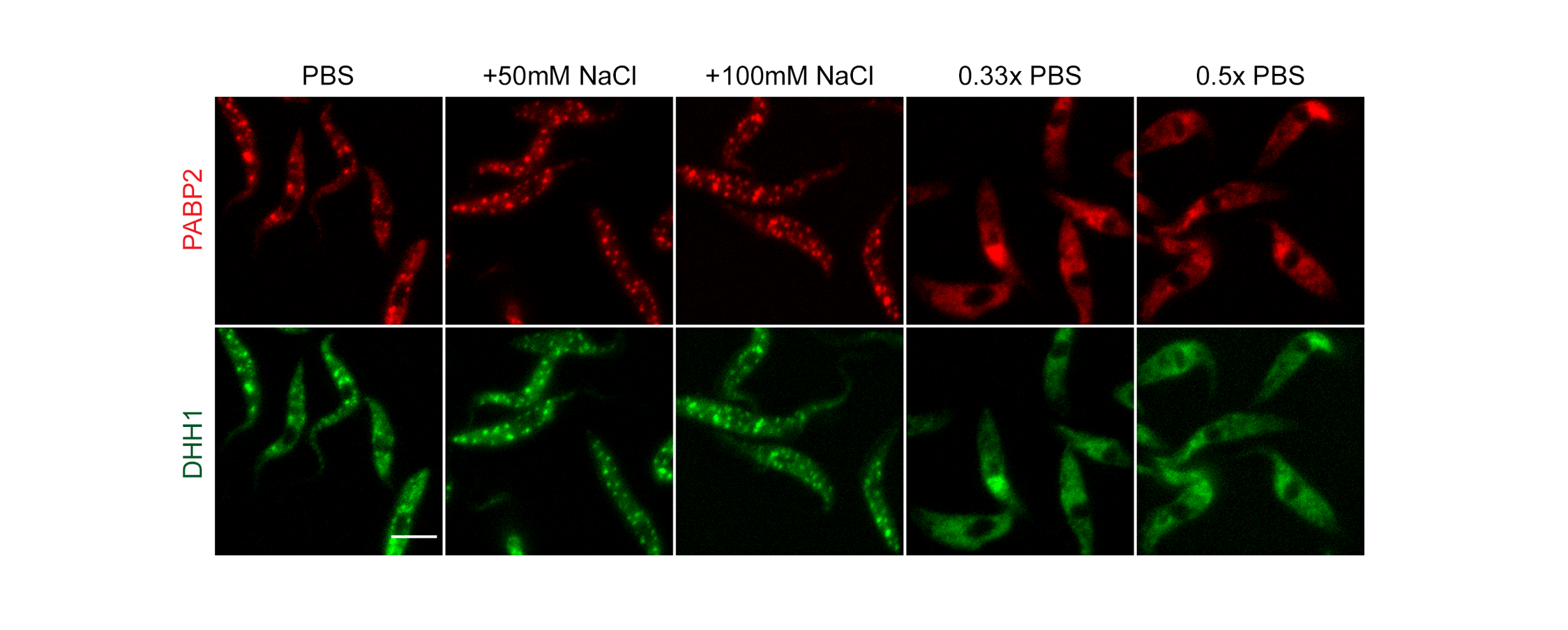

Supplement: S7 Fig — (TIF) [file ppat.1012666.s009.tif]
