# Supplementary material for: Dynamic composition of stress granules in Trypanosoma brucei
Source: PLoS Pathog. 2024 Oct 31;20(10):e1012666. doi: 10.1371/journal.ppat.1012666 (PMC11556693; doi:10.1371/journal.ppat.1012666)
Supplement: Attachment — Submitted filename: Response to comments.docx [file ppat.1012666.s010.docx]

Thank you very much for submitting your manuscript "Dynamic composition of stress granules in Trypanosoma brucei" for consideration at PLOS Pathogens. As with all papers reviewed by the journal, your manuscript was reviewed by members of the editorial board and by two expert independent reviewers. In light of the reviews (below this email), we would like to invite the resubmission of a significantly-revised version that takes into account the reviewers' comments.

Although both reviewers felt the paper had some interest, they also had substantial criticisms. One in particular Both were worried about cell viability, and one stated that at least some of the results might be due to a technical artefact - the fact that fluorescent proteins can be detected from within granules, whereas antibodies detect onl proteins on the outside (and therefore would also be less sensitive). Before this manuscript can be reconsidered it is thereofre essential that you address all of the questions raised by the reviewers. This includes providing the requested additional data on cell viability, and experiments in which the tags and antibodies are swapped between ALPH and PABP2. The possible technical issues must also be described and all additional methodological details requested provided. The Introduction needs to be re-written with clearer distinctions made between observations made in Opisthokonts and what is seen in trypanosomes.

We cannot make any decision about publication until we have seen the revised manuscript and your response to the reviewers' comments. Your revised manuscript will be sent to reviewers for further evaluation.

When you are ready to resubmit, please upload the following:

[1] A letter containing a detailed list of your responses to the review comments and a description of the changes you have made in the manuscript. Please note while forming your response, if your article is accepted, you may have the opportunity to make the peer review history publicly available. The record will include editor decision letters (with reviews) and your responses to reviewer comments. If eligible, we will contact you to opt in or out.

[2] Two versions of the revised manuscript: one with either highlights or tracked changes denoting where the text has been changed; the other a clean version (uploaded as the manuscript file).

Important additional instructions are given below your reviewer comments.

Please prepare and submit your revised manuscript within 60 days. If you anticipate any delay, please let us know the expected resubmission date by replying to this email. Please note that revised manuscripts received after the 60-day due date may require evaluation and peer review similar to newly submitted manuscripts.

Thank you again for your submission. We hope that our editorial process has been constructive so far, and we welcome your feedback at any time. Please don't hesitate to contact us if you have any questions or comments.

***********************

Your manuscript has been reviewed by two experts in the field and although both it had some interest, but also had substantial criticisms. Before this manuscript can be reconsidered it is essential that you address all of the questions raised by the reviewers. This includes providing the requested additional data on cell viability, and experiments in which the tags and antibodies are swapped between ALPH and PABP2 in order to be certain that the differences observed are not just a technical artefact caused by the fact that antibodies do not penetrate the granules. These technical issues must also be described and all additional details requested provided. The Introduction needs to be re-written.


Reviewer's Responses to Questions

**Part I - Summary**
Please use this section to discuss strengths/weaknesses of study, novelty/significance, general execution and scholarship.

Reviewer #1: In this work, the authors have looked into stress granules that they observed to form upon incubation of trypanosome cells in PBS or PBS with glucose, but not with PBS that contains proline and also not with PBS that contains physiological amounts of glucose.
The paper contains a range of novel observations: (i) PBS with low amounts of glucose (PBSg) causes SG formation, but PBS with proline does not (ii) PBS granules and gPBS granules have different features (ii) there are two “behaviors” of granule components, namely “PABP-like” and “DHH1-like”, referring on whether components would stay in the granules upon ATP depletion or not. (iii) granules have a substructure, with interior and exterior localized proteins.
These data give interesting insights into the types and dynamics of trypanosome stress granules. However, I do have concerns with this work. Many details that would be necessary for me to judge the conclusions are unfortunately missing. Some experiments miss important controls. I list the points below.

Reviewer #2: This manuscript from Aye and colleagues describes changes in protein composition of stress granules (SG) in the African trypanosome, Trypanosoma brucei in response to changes in cellular ATP levels. Through a variety of microscopic approaches after manipulation of environmentally available carbon sources, the authors describe dynamic changes in SG protein membership. Further, expansion microscopy and STED have revealed that SG components localize to different portions of the granules. These are new findings in trypanosomes; however, the connection between ATP and SG formation has been well-established for some time in other systems, reducing the potential overall impact of these findings.

**Part II – Major Issues: Key Experiments Required for Acceptance**
Please use this section to detail the key new experiments or modifications of existing experiments that should be absolutely required to validate study conclusions.

Generally, there should be no more than 3 such required experiments or major modifications for a "Major Revision" recommendation. If more than 3 experiments are necessary to validate the study conclusions, then you are encouraged to recommend "Reject".

Reviewer #1: Major points
• The introduction is too short and does not include any details on trypanosomes, about mRNA metabolism in trypanosomes, in particular, what is known and unusual about mRNA decay, translation, RNA granules. Translation initiation factors (why did the authors choose eIF4E1 rather than eIF4E3 in this work?)

Our response: Thanks for this suggestion. We have extended the introduction, adding more information on trypanosome mRNA metabolism that are relevant to this work.

Of the four eIF4E homologs, only eIF4E1 has been shown to colocalise with both PABP2 and DHH1 in stress induced granules of *Trypanosoma brucei* (1). Hence, eIF4E1 was used for analysis of granule localization and the “PABP2-like“ or “DHH1-like”behaviours.

• The same applies to Material and Methods: it lacks details on how the immunofluorescence was performed, which antibodies were used for the expansion microscopy and all other microscopy applications? How was the tri-marker cell line imaged, when it contains two fluorescent tags and one epitope tag? Expansion microscopy: what is the source of this protocol, that seems very different to standard protocols? How was the number of SG-containing cells quantified? (how do you define “SG-containing”?). Details on RNAi, image analysis, STED and Pearson’s coefficient are missing as well. It may seem not important on first view, which antibodies have been used and how exactly the imaging was done, BUT: the authors look into phase-separated areas which are restricted to antibody access, in particular this is true for the interior of trypanosome starvation stress granules, <https://ddec1-0-en-ctp.trendmicro.com:443/wis/clicktime/v1/query?url=https%3a%2f%2felifesciences.org%2freviewed%2dpreprints%2f95028%3a&umid=d629a389-40f9-46f7-ab7b-c7a95bc2b9b0&auth=8d3ccd473d52f326e51c0f75cb32c9541898e5d5-a0b0062b7751c0abab801f26b601a2bca5bc4170> IgG antibodies, in particular anti-HA, stain only outside of the granule, while fluorophores are detectable throughout the granules. Therefore, it is important to know exactly how the detection of each protein was performed.

Our response: Thanks for this important point. We have included more details on ExM and STED, the tags/antibodies, image analyses and the fixation/imaging conditions in the revised manuscript.

The ‘SG-containing’ cells are defined as cells with PABP2-positive puncta. This information is also included in the revised manuscript.

For expansion microscopy, we used a method (2-4) that allows localized antibodies to be linked to and expanded with the hydrogel, while the cellular proteins are cleared by protease treatments. In this method, direct fluorescence from the reporter proteins is destroyed upon protease digestion. Labelling of the target proteins is therefore achieved using anti-HA, anti-TY and anti-RFP (and secondary antibodies) prior to expansion. Compared to the UExM method that has been widely used for parasite studies (5), a main advantage of this protease-based method is its compatibility with all the antibodies/probes used for our routine immunofluorescence analyses and FISH analyses.

As suggested, we have also swapped the tags between ALPH1 and PABP2 and performed expansion microscopy (please see later section for details). The results are consistent with ALPH1 at the centre and PABP2 peripheral. This new result is included in new Fig. 5C.

• Line 188: Why would ALPH1 be a canonical PB marker, while DHH1 is not? Both ALPH1 and DHH1 localise to both PBs and SGs ... If at all, DHH1 is slightly “more canonical”, because it does not localize to the posterior pole and is an established P-body marker in yeast.

Our response: We agree with you. Both DHH1 and ALPH1 localize to organelles other than the PBs. We have modified the text accordingly.

• Cell death vs specific effect: Some experiments lack important controls, to test whether cells are still alive under the treatments, e.g. are the treatments reversible, when cells are retransferred into normal culture medium? One example is in Line 202 (another one is Figure 2D-F): ATP depletion by ED or 2DG prevents subsequent PB formation. How do the authors know, that these cells were still alive and not merely killed by the ATP depletion?

Our response: We have performed cell death assays on all starvation and ATP-depletion conditions (PBS, PBSG, ED, and PBS2DG). These results are included in the new Fig. S2.

• Figure 5: DHH1 RNAi. There are no information on how the RNAi was done (Material and methods or Figure legend). No controls for the success of the RNAi are shown or mentioned. No information on when these images were made (how long after induction of the RNAi?). The image quality is not good. It is not explained, how many cells were analysed and how exactly the Pearson’s coefficient was calculated (parameters).

Our response: We have decided to remove the part on DHH1 RNAi. DHH1 is essential for cell survival in culture (11). The function of DHH1 is likely not limited to PB/SG. The experiment shown in old Fig. 5 was performed 48 hours post RNAi induction and many cells have possibly died, and this may partly explain the poor morphology of the DHH1 RNAi cells.

• Expansion microscopy data: These data are very nice and its an interesting observation. However, the differences could also be caused by different abilities of the antibodies to penetrate to phase separated areas, see comment above. Therefore, it is essential to add these details. It is also essential to swap the tags and antibodies between ALPH and PABP2.
Our response: Thanks again for this great suggestion. Tags between ALPH1 and PABP2 were swapped. Expansion microscopy was conducted using anti-HA to visualize ALPH1-HA and anti-RFP for PABP2-mScarlet. Results are consistent with the previous experiment where PABP2-HA was labelled with anti-HA and ALPH1-mScarlet with anti-RFP. Hence, we rule out the possibility of differential antibody penetration.

Reviewer #2: 1. In Fig. 1A., the loss of ATP could result from cell death. While I appreciate that the literature supports that trypanosomes are viable in PBS for the study period, some evidence that they remain viable throughout the manipulation described here would be comforting.

Our response: Thanks for this suggestion. Cell death assays were conducted for all stress conditions used in this study (please see new Fig. S2D). The cell viability was not affected by different treatments.

2. In Fig. 1B, PABP2, ALPH1, and DHH1 in “Fed” cells “showed cytoplasmic distribution”. One possible explanation for this is that overall expression levels fall upon starvation, revealing foci of label. This should be assessed. Related, the text mentions what happens to the three markers at 30 min, but images are not provided. Please include those in Fig. 1B.

Our response: We have performed immunoblots, which showed that the overall protein levels of PABP2, ALPH1 and DHH1 were not affected upon PBS and gPBS starvation. Puncta formation is thus likely due to protein redistribution and phase separation of cytoplasmic proteins into SG condensates. Our results are also consistent with a previous study (12), where the overall expression level of PABP2 and DHH1 was found unchanged before and after PBS starvation. We have included the immunoblots in revised Fig. S1.


3. Fig. 1 C/D, but others also (Fig. 2D/F, Fig. 5B, Fig. 7B) – The legends need to include information about how many cells/ fields of cells were used to calculate the data used in these graphs to support the rigor of the work.

Our response: Thank you for the suggestion. We have included the cell number information (at least 200 cells were counted for each condition) in the figure legends.

4. The experiments describing sub-granule localization need some clarification. First, the demonstration that PABP2 is in SGs by expansion microscopy is supported by co-localization with mRNA (in a supplemental figure). This localization is different (qualitatively – less punctate, fewer individual granules) than co-labeling with other markers. While I appreciate that the SGs are organized based on LLPS and therefore are inherently subject to possible morphological changes due to sample handling, is it possible that the granules described throughout this work are a mix of SGs and some other type of granule? The STED images of dT co-localization also has structures that do not label with dT. Some discussion of this issue is warranted.

Our response: mRNA labelling by dT was done using a fluorescence in situ hybridization protocol (please see the materials and methods for details). The pre-hybridization and wash steps are different to immunofluorescence labelling and possibly harsher to the cells. This may explain the less punctate, fewer granules morphology of PABP2-containing SGs observed in dT-labelled cells. For STED imaging, as we had to use Alexa 568-streptavidin instead of an Abberior Star Red probe, the dT labelling was not as bright as in regular immunofluorescence microscopy or ExM. This may explain the weak dT labelling in some PABP2-granules in the STED images. Thank you so much for pointing this out, and we have included discussion on this issue in the revised text.

5. The expansion images should include a brightfield image to allow assessment of cell quality. Please also include some indication of the percentage of granules that have the PABP2 labeling phenotype (hollow region at center or off to one side).

Our response: We used an expansion microscopy method (2) that allowed localized antibodies to be linked to (and expanded with) the hydrogel, while the cellular proteins were cleared by protease treatments. As such, brightfield image of the cells cannot be obtained. Upon analysis of more than 100 gPBS-induced granules, ~80% of the granules have PABP2-granules showing hollow regions at the centre or off to one side. It is possible that the remaining granules did not develop any hollow regions or their orientation did not allow the hollow region to be detected. This information is included in the revised text.

**Part III – Minor Issues: Editorial and Data Presentation Modifications**
Please use this section for editorial suggestions as well as relatively minor modifications of existing data that would enhance clarity.

Reviewer #1: • Figure 3 and S4: I would prefer to see the S4 data in the main Figures, as this is a key finding.

The S4 data has been moved to new Figure 3.

• Figure S5 is a major finding on the difference between PBS and gPBS granules, best not to hide in supplementary figures. The data show major differences in granule stability, that are very interesting.

The data has been moved to new Figure 5.

• For DEAD box RNA helicases, the ATP is in general needed to release the enzyme from the RNA, rather than for the helicase activity itself, please  include this in the discussion -  why, then, should lack of ATP cause Dhh1 to be released from the SGs? (You would expect the opposite.)

It has been shown that ATP binding of DHH1 is required for its assembly into granules (PBs), whereas the ATPase activity is required for disassembly/release of the granules (13). As such, the cycle of ATP binding and hydrolysis allows the dynamic behaviour of DHH1. Upon ATP depletion during starvation, this dynamic recycling DHH1 is likely compromised, resulting in their cytoplasmic localisation. We have included this in the revised discussion.

• Line 306: “Indeed, DHH1 interaction with both ALPH1 and PABP2 is observed in Cary 2015 and Kramer 2023)”. Cary 2015 is a paper about DHH1 in yeast, which has no homologue to ALPH1. Same on line 373: ALPH1 is specific to Kinetoplastida and not present in yeast.

These sentences have been removed or revised in the text.

Reviewer #2: 1. Minor, but the graphs in Fig. 2A and B should be switched – PBS starvation is mentioned first in Fig. 1, so to be consistent it should likewise be first here.
2. Line 16, “remove the semicolon
3. Line 20, change “causing” to “that causes”
4. Line 35, add “the” before “bulk”
5. Line 39, remove the comma
6. Add “the” before “novel”
We have included all above changes.

**References**

1.	Kramer S, Bannerman-Chukualim B, Ellis L, Boulden EA, Kelly S, Field MC, et al. Differential Localization of the Two T. brucei Poly(A) Binding Proteins to the Nucleus and RNP Granules Suggests Binding to Distinct mRNA Pools. Preiss T, editor. PLoS ONE. 2013 Jan 30;8(1):e54004.

2.	Chozinski TJ, Halpern AR, Okawa H, Kim HJ, Tremel GJ, Wong ROL, et al. Expansion microscopy with conventional antibodies and fluorescent proteins. Nat Methods. 2016 Jun;13(6):485–8.

3.	Chen L, Yao L, Zhang L, Fei Y, Mi L, Ma J. Applications of Super Resolution Expansion Microscopy in Yeast. Front Phys. 2021 Mar 29;9:650353.

4.	Hawkins TJ, Robson JL, Cole B, Bush SJ. Expansion Microscopy of Plant Cells (PlantExM). In: Hussey PJ, Wang P, editors. The Plant Cytoskeleton [Internet]. New York, NY: Springer US; 2023 [cited 2024 Sep 12]. p. 127–42. (Methods in Molecular Biology; vol. 2604). Available from: https://link.springer.com/10.1007/978-1-0716-2867-6_10

5.	Kalichava A, Ochsenreiter T. Ultrastructure expansion microscopy in *Trypanosoma brucei*. Open Biol. 2021 Oct;11(10):210132.

6.	Luo Y, Na Z, Slavoff SA. P-Bodies: Composition, Properties, and Functions. Biochemistry. 2018 May 1;57(17):2424–31.

7.	Wilczynska A, Aigueperse C, Kress M, Dautry F, Weil D. The translational regulator CPEB1 provides a link between dcp1 bodies and stress granules. Journal of Cell Science. 2005 Mar 1;118(5):981–92.

8.	Newbury SF, Mühlemann O, Stoecklin G. Turnover in the Alps: an mRNA perspective: Workshop on Mechanisms and Regulation of mRNA Turnover. EMBO Reports. 2006 Feb;7(2):143–8.

9.	Stoecklin G, Kedersha N. Relationship of GW/P-Bodies with Stress Granules. In: Chan EKL, Fritzler MJ, editors. Ten Years of Progress in GW/P Body Research [Internet]. New York, NY: Springer New York; 2013 [cited 2024 Jan 22]. p. 197–211. (Advances in Experimental Medicine and Biology; vol. 768). Available from: https://link.springer.com/10.1007/978-1-4614-5107-5_12

10. Kramer S. The ApaH-like phosphatase TbALPH1 is the major mRNA decapping enzyme of trypanosomes. Hill KL, editor. PLoS Pathog. 2017 Jun 19;13(6):e1006456.

11. Kramer S, Queiroz R, Ellis L, Hoheisel JD, Clayton C, Carrington M. The RNA helicase DHH1 is central to the correct expression of many developmentally regulated mRNAs in trypanosomes. Journal of Cell Science. 2010 Mar 1;123(5):699–711.

12. Fritz M, Vanselow J, Sauer N, Lamer S, Goos C, Siegel TN, et al. Novel insights into RNP granules by employing the trypanosome’s microtubule skeleton as a molecular sieve. Nucleic Acids Res. 2015 Sep 18;43(16):8013–32.

13. Mugler CF, Hondele M, Heinrich S, Sachdev R, Vallotton P, Koek AY, et al. ATPase activity of the DEAD-box protein Dhh1 controls processing body formation. eLife. 2016 Oct 3;5:e18746.
