# Supplementary material for: Dynamic composition of stress granules in Trypanosoma brucei
Source: PLoS Pathog. 2024 Oct 31;20(10):e1012666. doi: 10.1371/journal.ppat.1012666 (PMC11556693; doi:10.1371/journal.ppat.1012666)
Supplement: Attachment — Submitted filename: Reviewer responses.docx [file ppat.1012666.s011.docx]

**Part I - Summary**
Please use this section to discuss strengths/weaknesses of study, novelty/significance, general execution and scholarship.

Reviewer #1: The authors have addressed all the issues I had, in particular, the switch of the antibodies was very important and it's nice to see that it has worked and supports the findings. It is a very nice paper and I recommend publication.

Reviewer #2: This manuscript from Aye and colleagues describes changes in protein composition of stress granules (SG) in the African trypanosome, Trypanosoma brucei in response to changes in cellular ATP levels. Through a variety of microscopic approaches after manipulation of environmentally available carbon sources, the authors describe dynamic changes in SG protein membership. Further, expansion microscopy and STED have revealed that SG components localize to different portions of the granules.

**Part II – Major Issues: Key Experiments Required for Acceptance**
Please use this section to detail the key new experiments or modifications of existing experiments that should be absolutely required to validate study conclusions.

Generally, there should be no more than 3 such required experiments or major modifications for a "Major Revision" recommendation. If more than 3 experiments are necessary to validate the study conclusions, then you are encouraged to recommend "Reject".

Reviewer #1: (No Response)

Reviewer #2: The authors have largely addressed my concerns. One that was not addressed – (from initial review, related to Fig. 1B) “In Fig. 1B, PABP2, ALPH1, and DHH….the text mentions what happens to the three markers at 30 min, but images are not provided. Please include those in Fig. 1B.” If the data is going to be mentioned, it should be included. Alternatively, the text could be altered to compare 0.25 h in the starved to 0.5 h in the gPBS, I think the conclusions would not change dramatically.

Our response: Sorry for overlooking this point. We have revised the text (line 236) to remove the reference to the 0.5 h images.

**Part III – Minor Issues: Editorial and Data Presentation Modifications**
Please use this section for editorial suggestions as well as relatively minor modifications of existing data that would enhance clarity.

Reviewer #1: (No Response)

Reviewer #2: 1. Author summary. Line 37, “In the single cellular Trypanosome parasites” to “In the single-celled trypanosome, SGs”
2. Line 74 – add “the” between “at” and “post-transcriptional”
3. Line 78. End the sentence after eIF4G. Then , “Additionally,….”
4. Line 369 – change “is” to “was”

Our response: All of the above have been changed in the revised manuscript.
